# Biblio-MetReS: A bibliometric network reconstruction application and server

**DOI:** 10.1186/1471-2105-12-387

**Published:** 2011-10-05

**Authors:** Anabel Usié, Hiren Karathia, Ivan Teixidó, Joan Valls, Xavier Faus, Rui Alves, Francesc Solsona

**Affiliations:** 1 Department d'Informàtica i Enginyeria Industrial, Universitat de Lleida, Av. Jaume II n°69, 25001 Lleida, Spain; 2 Department de Ciències Mèdiques Bàsiques & IRBLleida, Universitat de Lleida, Montserrat Roig n°2, 25008 Lleida, Spain

## Abstract

**Background:**

Reconstruction of genes and/or protein networks from automated analysis of the literature is one of the current targets of text mining in biomedical research. Some user-friendly tools already perform this analysis on precompiled databases of abstracts of scientific papers. Other tools allow **expert **users to elaborate and analyze the full content of a corpus of scientific documents. However, to our knowledge, no **user friendly **tool that simultaneously analyzes the latest set of scientific documents available on line and reconstructs the set of genes referenced in those documents is available.

**Results:**

This article presents such a tool, Biblio-MetReS, and compares its functioning and results to those of other user-friendly applications (iHOP, STRING) that are widely used. Under similar conditions, Biblio-MetReS creates networks that are comparable to those of other user friendly tools. Furthermore, analysis of full text documents provides more complete reconstructions than those that result from using only the abstract of the document.

**Conclusions:**

Literature-based automated network reconstruction is still far from providing complete reconstructions of molecular networks. However, its value as an auxiliary tool is high and it will increase as standards for reporting biological entities and relationships become more widely accepted and enforced. Biblio-MetReS is an application that can be downloaded from http://metres.udl.cat/. It provides an easy to use environment for researchers to reconstruct their networks of interest from an always up to date set of scientific documents.

## Background

Reconstructing molecular networks that are responsible for regulating biological processes is a fundamental task in molecular biology, if one is to understand how the different components of those networks contribute to each process. In recent years many alternative types of methods have been proposed to achieve such a reconstruction [1,2]. One type of method relies on the automated analysis of published literature to identify genes and proteins that co-occur in the same document(s) [3-11]. It has been assumed that if two genes or proteins are cited in the same document, there is the likelihood that they functionally interact. In fact, many algorithms, methods and tools have been proposed and implemented in order to reconstruct the network of genes associated with a given gene of interest, by automated mining of the published literature [3-31].

Only a small number of these tools are more widely cited (and likely used) by molecular biologists (Table 1). Out of these, iHOP [3] and STRING [5] have a usage that is at least one order of magnitude higher than that of other applications ^1^, as estimated by the number of times that the different applications are cited (Table 1). These two web servers preprocess documents that are published in Medline and PubMed, looking for words that match the names of genes from the different organisms in the web server's database. Once they have identified the genes that co-occur in those documents, they provide different functionality to the user. While iHOP allows the user to choose exactly which genes s/he wants to add to the interaction network, STRING automatically establishes a threshold score above which all genes are included in the model for the network.

**Table 1:** Number of citations for text mining programs in the Web of Science database as of June 2011

Program	Total Number of Citations
STRING	949
iHOP	274
Whatizit	41
Alibaba	37
Reflect	16
iProLink	11
SciMiner	4
BioLMiner	1
Linguamatics I2E	1
Akane RE	0
Laitor	0
PathText	0

A shortcoming of both these tools is that, in terms of literature, they only analyze the information contained in Medline or PubMed abstracts and their databases require constant update. Given that policies for publication and access to scientific papers are changing and, as a consequence, an increasing number of scientific publications are becoming freely available over the internet, iHOP and STRING ignore a growing source of information about possible interactions between genes [20,32,33].

Currently, other tools that analyze full documents without pre-processing in order to reconstruct molecular gene networks are either still experimental, applicable only to a document or documents supplied by the user or present in PubMed [6,9,11,34,35] and/or require a high level of computational expertise for their use [6,34,35].

Thus, there is a need for a tool that a) analyzes full documents as they are made available on the world wide web and before they are included in databases such as PubMed, b) analyzes documents and literature corpora that have not been manually annotated, and c) is user-friendly. We developed Biblio-MetReS http://metres.udl.cat/ to meet these demands, allowing for an on-the-run full text analysis for automated reconstruction of literature gene/protein networks in an intuitive way. Biblio-MetReS relies on a database that contains lists of all annotated genes of organisms with fully sequenced genomes from the KEGG database. The tools allows users to select different sources of information from where to compile data for the reconstruction of the molecular networks responsible for regulating and executing biological processes.

Here we present the tool and benchmark it against STRING and iHOP, using genes that participate in well characterized metabolic processes of organisms with fully sequenced genomes. The three tools have comparable results when Biblio-MetReS searches are limited to Medline. When this limitation is removed, Biblio-MetReS finds networks that are more complete than those found by iHOP and STRING.

## Implementation

### Underlying database & Biblio-MetReS implementation

Biblio-MetReS relies on an in-house database of organisms and genes that was built using the list of organisms with fully sequenced genomes available in KEGG [36]. The database of gene names and their synonyms is built and regularly updated by matching the KEGG gene names and synonyms to their NCBI [37] names and synonyms, followed by removing of redundant terms. The databases are implemented using Zope technology, which is based on MySQL and Python.

The application itself was implemented in JAVA, using the NetBeans IDE. Swing was used to implement the Graphical User Interface (GUI). Swing was also used to create the parsers for the different documents to be analyzed, with the exception of PDF files. These files are parsed using the PDFBox library. We implemented parsers for HTML documents, PDF and ASCII. HTML documents are transformed into plain text as follows: paragraphs are detected in the HTML code, using a parsing library to navigate through the tags, followed by extraction of the text within those tags. PDF documents are transformed into plain text using the Pdfbox library, which extracts the text within the document while ignoring the images. Once the text is extracted, we parse for paragraphs by looking for punctuation signs that signal the end of a sentence followed by the new line escape character. These punctuation signs are used to split sentences, controlling to make sure that we are not splitting decimal figures, e-mail addresses, web pages, and others.

The results are stored in a file with XML format that is generated at the end of each search. The processing of the XML files is done using the JDOM API. The JGraph API is used for the graphical representation of the network results in 2D.

### Document search and analysis

**Figure 1:**
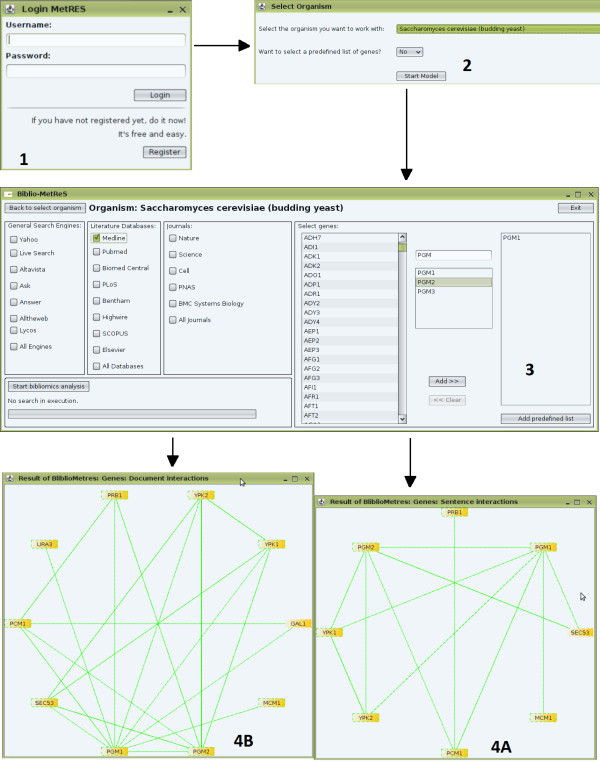
**Workflow of Biblio-MetReS**. The user registers, logs in (Panel 1) and selects an organism (Panel 2). Once selected, the program either loads the full list of genes from which the user will select the genes to analyze (Panel 3) or allows the user to directly insert the genes s/he wants to analyze (Panel 2.1). Then, the user must select the databases and web searchers s/he wants to use (Panel 3). The program then starts the search and when finished, it generates a series of outputs for the results. First, the list of documents that was analyzed is shown, together with links to the document and to the list of genes fount in each document (Panel 4A). Second, a list of all genes that were found is given (Panel 4B). Each gene is linked to its KEGG webpage, where the gene is associated with other databases and biological pathways. Third, a tabular analysis of gene co-occurrence is shown (Panel 4C). This table contains access to information about the documents and sentences where gene pairs are found (Panels 4C.I and 4C.II). Finally, two editable graphs containing the sentence (Panel 5 A) and paragraph (Panel 5 B) co-occurrence networks are shown.

Biblio-MetReS implements a meta-search engine that compiles results from the search engines selected by the user (see Figure 1, panel 3 for a list of document sources). The search that is launched to each search engine includes all genes selected by the user, as well as the name of the organism of interest. As the search is completed by the relevant search motor (or motors if the user selected more than one data source), Biblio-MetReS collects the URLs of all documents found by each of the search engines. The treatment of these URLs goes as follows. First, the application eliminates redundant URLs. Then, for results from scientific databases and journals, it analyzes the *doi *number for each document, eliminating further duplicates. When the non-redundant list of documents is ready, Biblio-MetReS identifies if the full text of the document is freely available (either because the text is free to all users or because the institution providing the web connection has access agreements with the content provider) or if it is protected. In the latter case, the application discards this document and analyzes only the freely available abstract. Once all this pruning procedure is done, the application analyzes each document in search of co-occurrence of any genes or proteins in sentences, paragraphs and entire documents. Exact name matching is used and all synonyms for a gene are searched for. The dictionary of synonyms we use is a merge from those of NCBI and KEGG.

The analysis of co-occurrence in sentences and paragraphs is done because, when analyzing the full text of scientific documents, one must consider some form of proximity measurement. Otherwise, the co-occurrence of genes in different sections of the same document will introduce a significant amount of noise in the network of possible interactions [20].

### Metrics

We need to define appropriate metrics in order to provide some degree of biological significance to the fact that if two genes or proteins co-occur in a document they do not do so by pure chance. To do this we consider different aspects of co-occurrence.

First, we measure how frequently the different proteins or gene pairs co-occur in sentences, paragraphs and/or documents. We then take the odds ratio of the frequency of occurrences in the first two categories with respect to that of the third. The closer to one these odds ratios are, the more frequent it is that both genes are mentioned only in the same sentences or paragraphs of a document, rather than appearing haphazardly in different sections of the text.

Second, we calculate how much information we gain by having the two genes co-occur, when compared to the individual occurrences of the two genes. To estimate this we use information theory. The individual probability of occurrence of a gene is denoted as p(Gi) and it is formally defined as $p\left(Gi\right)=\frac{a}{n}$, where **a **is the number of documents where gene *i *appears, and **n **is the total number of documents.

The joint probability of co-occurrence of two genes, p(Gi, Gj), is defined as $p\left(Gi,Gj\right)=\frac{b}{n}$, where **b **is the number of documents where genes *i *and *j *simultaneously appear, and **n **is the total number of documents.

Having defined how to calculate the various probabilities, the mutual information, MI(Gi, Gj), is calculated as follows:

$$MI\left(Gi,Gj\right)=p\left(Gi,Gj\right)log\left(\frac{p\left(Gi,Gj\right)}{p\left(Gi\right)p\left(Gj\right)}\right)$$

where the applied logarithm is in natural base.

Finally, and in order to attribute some form of statistical significance to the co-occurrence of a pair of genes, we analyze contingency tables for those co-occurrences. The analysis is as follows. Consider a set of n sentences (paragraphs, documents) [1 ..., n]. For a given gene k define

$${\text{y}}_{\text{ik}}=\left\{\begin{array}{c} \begin{array}{ccc} 1 & ⇐ & \begin{array}{c} gene\;k\;occurs\;in\;sentence\\ \left(paragraph,\;document\right)\;i \end{array} \end{array}\\ \begin{array}{ccc} 0 & ⇐ & otherwise \end{array} \end{array}\right)$$

Now, for genes k_1 _and k_2 _define

$$\phi_{k1,k2}=y_{i,k1}y_{i,k2}$$

which has value 1 when both genes co-occur and 0 otherwise.

Both these variables have a Bernoulli distribution. If the occurrence of genes k1 and k2 is independent, then p(ϕ_k1, k2_) = p(y_k1_) p(y_k2_) would be expected, where p(y_k·_) is the relative frequency of occurrence of gene y_k· _and p(ϕ_k1, k2_) is the relative frequency of co-occurrence of genes k_1 _and k_2 _in the total number ***n ***of sentences (paragraphs, documents). Then, a Pearson statistic can be used to test for independence of occurrence between k_1 _and k_2 _by comparing the observed frequencies, n_1 _= ***n ***p(ϕ_k1, k2_) and n_2 _= (1-p(ϕ_k1, k2_))***n***, with the expected frequencies under the null hypothesis of independence, which would be m_1 _= ***n ***p(y_k1_) p(y_k2_) and m_2 _= ***n ***(1-p(y_k1_) p(y_k2_)). The Pearson statistic is computed as follows

$$X^{2}=\sum_{i=1}^{2}\frac{{\left(n_{i}-m_{i}\right)}^{2}}{m_{i}}$$

This statistics follows a chi-square distribution with one degree of freedom, i.e. $\chi_{1}^{2}\sim X^{2}$; hence, the p-value can be calculated as $p=Pr\text{(}\chi_{1}^{2}>\chi^{2}\text{)}$ to assess whether the observed co-occurrence is higher than the one expected by pure chance.

## Results

### The workflow

Figure 1 summarizes the workings of Biblio-MetReS. For security reasons users need to register before their first use, in order for the application to be able to access the central database. Once they have registered and logged in, an organism is chosen to work with. The application loads all genes from this organism that are present in the central database. Once the loading is finished, the user is presented with a window where s/he has to select the data sources for the analysis as well as the genes that will start the analysis. There are three types of data sources to choose from: General Engines (Yahoo, ...), Literature Database (Medline, ...) and Journals (Nature, ...). Once the choices are made and the search is started, the tool identifies the documents that contain the gene names provided by the user and their synonyms. Then, it extracts the full text from each document, and analyses for the co-occurrence of any pair of genes from the organism. All this processing is done on the fly.

The results of the analysis are presented to the user in several forms (Figure 1). First, Biblio-MetReS provides identifying information about each document that it analyzed, together with a list of links to those documents. If the user clicks on any of these links, the documents will open in their default browser. The user is also provided with a list of all genes and gene pairs that were found in each document.

Second, Biblio-MetReS presents the results of co-occurrence as tables. In these tables, the program provides information about absolute and relative frequencies of gene co-occurrence, linked to mutual information and p-values. The tables also provide links to gene and pathway information from other databases.

Third, the results are also presented as two graphs. These graphs provide alternative representations of co-occurrence. One graph presents the co-occurrence of genes in sentences, while the other presents the co-occurrence of genes in paragraphs and documents. In these graphs, each node or vertex is a gene/protein and each edge refers to the interaction between genes/proteins. The thickness of the edge is proportional to the mutual information between two genes and the colour of the edge is proportional to the p-value for the co-occurrence between the two genes or proteins. The colour scale changes in a continuous manner between red (non-significant) and green (significant).

### Comparing Biblio-MetReS to iHOP and STRING

Given that Biblio-MetReS is intended for an audience similar to that of iHOP and STRING, we need to compare how the results of the three tools differ amongst each other. To do this, we selected three pathways described in KEGG for four different organisms (Additional File 1). In each organism, and starting from a set of three or four genes per pathway, we performed a network reconstruction for each of the three pathways under different conditions (Additional File 1).

iHOP and STRING only search Medline or PubMed abstracts that are pre-processed and stored internally by each program. Because of this, a comparison between the results of these applications and those from Biblio-MetReS require that the set of documents analyzed by Biblio-MetReS is restricted to those contained in Medline. Furthermore, because Biblio-MetReS always analyzes the most recent update of Medline at NCBI, it was run to analyze only the 20 most relevant abstracts from Medline, to avoid an unfair advantage. Our analysis led to the following observations.

**Figure 2:**
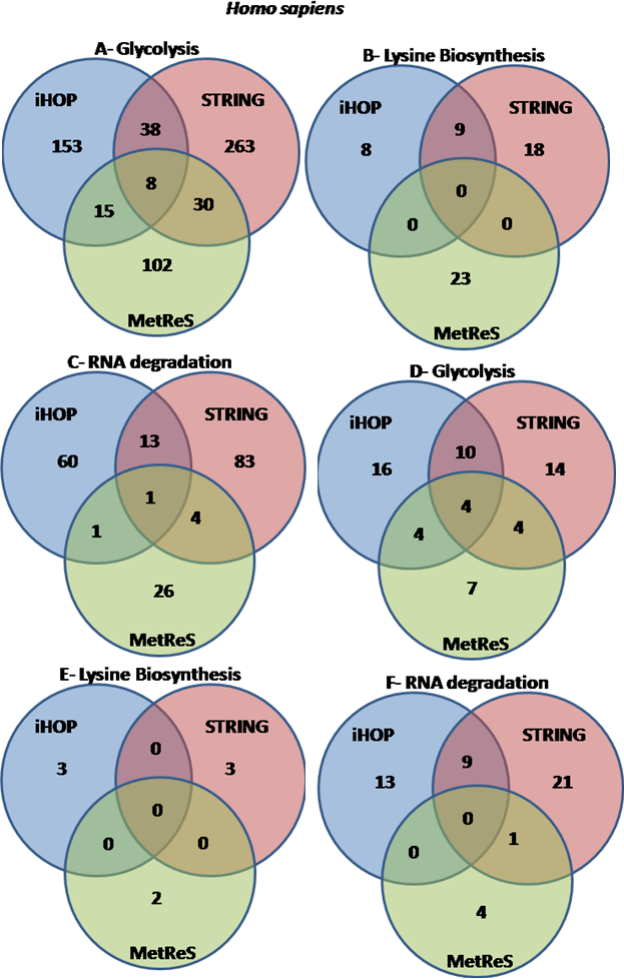
**Comparison of results between Biblio-MetReS, iHOP and STRING**. Representation of the number of common genes found for the different pathways in *Homo sapiens *using Biblio-MetReS, iHOP and STRING. This figure shows all genes found for each test. Additional File 3 shows the results for the other organisms, as well as for the genes that are not considered to be in the canonical pathways. A - Glycolysis, all genes. B - Lysine metabolism, all genes. C - RNA degradation, all genes. D -. Glycolysis, only genes known to belong to the canonical pathway. E -Lysine metabolism, only genes known to belong to the canonical pathway. F - RNA degradation, only genes known to belong to the canonical pathway.

First, Biblio-MetReS, iHOP and STRING generate different results, even though the literature corpus that they analyze is, in principle, the same (Figure 2, Additional File 2). This is likely to be the result of a) different processing of PubMed abstracts (either because the two tools update their databases at different times or because they process abstract content differently), and b) dictionaries that provide synonyms to the standard gene names that do not fully overlap in each of the three tools. In particular STRING uses internal pre-compiled synonym dictionaries, iHOP uses Entrez Genes, FlyBase, UniProt and the classification from the HUGO nomenclature Committee, and Biblio-MetReS uses KEGG, UniProt and NCBI nomenclature. We cannot control or further investigate a), as this would require access to the inner workings of each program. However, we controlled for b) by checking by hand if all genes we found in one dataset had synonyms in the other two or not, but many of the differences remained (Figure 2).

Second, even with the self-imposed limitation of using only the 20 more relevant abstracts, Biblio-MetReS always found a number of genes that is comparable to that found by either iHOP or STRING (Figure 2, Additional File 2).

Third, and as a way to control for the quality of the result from each program, we analyzed how many of the genes that are found by each application are known to be a part of the pathways, as defined in KEGG. No applications find all genes that are associated with the different pathways. In fact, only between 5% and 30% of all genes that were found by the three applications are annotated in KEGG as being a part of the relevant canonical pathway. The application that finds the largest number of genes associated with a canonical pathway varies and is case-dependent (compare Additional Files 2 and 3). No single application performs best neither in all pathways of a given organism nor in all organisms for a single pathway. In addition, all application finds several genes that are not associated with the canonical KEGG pathways but co-occur with pathway genes in the literature. In fact between 70% and 95% of all genes identified by iHOP, STRING, or Biblio-MetReS belong to this category. This reveals one of the benefits of these applications, that of finding associations that are not commonly considered. However, this benefit is also associated with the risk of misidentification of functionally interacting genes (see below).

### Contribution of the different data sources

Given that one of the added values of Biblio-MetReS is its capacity to search and analyze full text documents, we tested how different sources of information added to the number of genes that were found. In these tests, we use the different types of source information ("Literature Databases", "Journals" and "General Engines") in order to find out how much information the different sources add to the reconstruction process. Additional File 1 contains a summary of the tests performed for this analysis.

First, our results suggest that using general search engines for this type of network reconstruction should be done sparingly, if at all. In every test case these engines found files with the entire fully annotated set of genes from the relevant organism. This means that the sensitivity of these search engines for the job of finding co-occurring genes in documents is very high. However, their selectivity is null. Therefore, we do not recommend using these engines when reconstructing a gene network. Because of this we performed the remainder of the benchmark tests using only the search engines from the Literature Databases and Journals panes of Biblio-MetReS (see Figure 1 panel 3).

**Figure 3:**
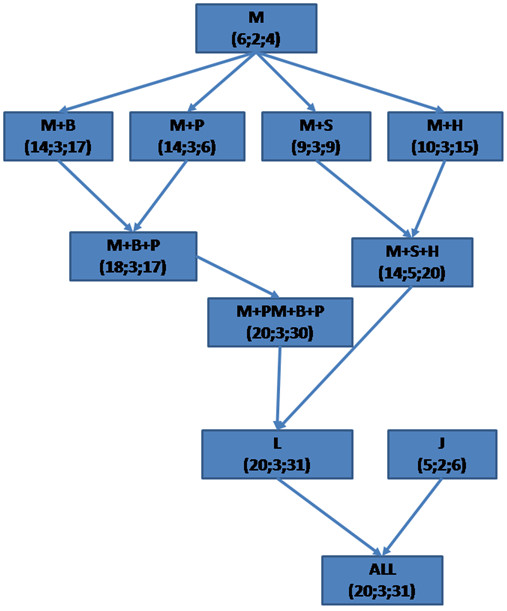
***Homo sapiens*: Representation of the number of additional genes found by Biblio-MetReS that are known to belong to the canonical pathways under analysis, as we add more data sources to Medline**. Each panel shows three numbers in each square. The first number represents the number of genes found for glycolysis. The second number shows the number of genes found for lysine metabolism. The third number shows the number of genes found for RNA degradation.

Second, we compared the sensitivity of Biblio-MetReS using different databases for scientific documents (Figure 3 and Additional Files 4 and 5). In general, Medline is the database in which a smaller number of genes is found. When Medline analysis is compared to analysis of databases containing the full text of scientific papers from individual journals or publishing houses, more genes that belong to the relevant pathways are almost always found in the latter case. This suggests that, many times, the information gain provided by analyzing the full text of scientific papers of a given publisher more than offsets the loss of information caused by only having access to a fraction of the scientific literature.

Nevertheless, as is the case when comparing iHOP, STRING and Biblio-MetReS (using Medline), each literature database generates a set of genes that, in many cases, is only partially overlapping. Therefore, we analyzed how much is gained by combining the different literature sources. Additional Files 4 and 5 summarize the results of this analysis.

We find that, in general, searching the set of individual journals that we include in Biblio-MetReS discovers a smaller number of gene interactions than using Medline. We also find that, as we combine larger databases, the number of genes that belong to the network of interest increases. However, so does the number of genes that are not recognized by KEGG as being associated with the pathway. In general, a search in literature databases identifies all the genes that are also identified when searching the set of individual journals. However, in some cases, the sets of genes found in the two types of databases are absolutely complementary. This is the case of the genes for glycolysis in *Drosophila melanogaster*.

Another aspect of interest that needs to be analyzed is that of discrimination between genes that are known to belong to the different canonical pathways under analysis and genes whose association to those of the pathway is indirect. Additional File 4 shows how many of the genes found by Biblio-MetReS are annotated as belonging to the relevant pathways in KEGG. For example, compare the squares marked M (Medline) in each panel of Additional File 4 to the subsequent squares in the same panel. You can see that Biblio-MetReS now finds between 1.5 and 6-7 times more genes associated with the canonical pathway than any of the applications in benchmark 1. In contrast, Additional File 5 shows the total number of genes found during the analysis. We find that most of the genes that are found by the program in the different combinations of databases are not directly associated with the canonical pathway being tested. This was also the case in the first benchmark tests for the three applications being compared (Biblio-MetReS, iHOP, and STRING). The percentage of the total genes that are outside the canonical pathway increases with the number of documents being analyzed.

One way to filter many of the interactions with additional genes that may be irrelevant is by analyzing the graph of genes that co-occur in sentences. The sentence co-occurrence network has a much smaller number of interactions between genes (compare panels 5A and 5B in Figure 1). These interactions are enriched in interactions between genes that belong to the canonical pathway. Furthermore, it is easier for the user to identify if a gene association in this network is important for the work at hand, because Biblio-MetReS shows the relevant sentences.

## Discussion

Automated text mining efforts with the goal of extracting biological information is a booming field. Many issues still need to be solved in order for this extraction to be as good as it can be. On one hand, reporting of biological entities and concepts still needs to be standardized and standards need to be fully accepted and implemented by both journals and researchers. On the other, more efficient methods also need to be developed. The BioCreAtIvE challenge has been established to evaluate how well the different methods perform in both identifying biological entities and relationships between these entities [38].

The BioCreAtIvE challenge, as any control experiment should do, performs an evaluation of different tools in well curated datasets. However, while more developed methods are being further developed, biological researchers can still benefit from prototypical applications that assist them in many the large majority of the scientific literature, which is not curated at all. Efforts to mine this body of literature in order to reconstruct networks of interacting genes started as early as in the end of the nineties [39]. In the first decade of the twenty first century, a few tools have been developed to enable this reconstruction. Most of these require a non-trivial amount of computational knowledge if they are to be used. Some, such as iHOP and STRING, are widely used and user-friendly. Each of these applications searches a database of scientific documents that was previously analyzed and processed. This pre-processing strategy makes the identification of co-occurring genes a faster process at the cost of disregarding documents present in PubMed and/or Medline but not yet processed by the pipeline underlying the applications. Biblio-MetReS, which is developed to fit in this user friendly category, provides the following added value with respect to iHOP and STRING:

1 - Our reconstruction is done live and with the latest available documents on the internet. In contrast, iHOP and STRING use a precompiled database of documents for their search. This means that our results will be more up to date than those of the other two applications.

2 - While iHOP, STRING, and Biblio-MetReS search for gene interactions in abstracts of Medline and PubMed documents, Biblio-MetReS can additionally search full documents from other scientific and general data sources. This increases the number of gene associations that can be found. Nevertheless, it has been reported that the analysis of complete scientific documents may increase the noise in gene associations that are found [20,32].

3 - A third additional functionality provided by Biblio-MetReS with respect to iHOP and STRING permits filtering out some of the noise that may arise from the analysis of complete documents. Our tool distinguishes between co-occurrence of genes and proteins in sentences, paragraphs and whole documents. The analysis of sentences decreases the probability of detecting spurious associations between genes that are found in different parts of the documents and may have little to do with one another.

Both pre-processing of documents strategies, as done by iHOP and STRING, and on-the fly analysis strategies, as done by Biblio-MetReS or Reflect, have disadvantages. This first strategy has the cost of using information that is almost never quite up to date, while the latter has the cost of becoming potentially very slow. One way to side-step these disadvantages is by combining both strategies in the same tool. We are working on an implementation of Biblio-MetReS that will do this. In fact, the next version of Biblio-MetReS is being implemented in such a way that the results of each search will be stored and compiled. Thus, if a new search finds a document that has been analyzed before, it will retrieve the processed data from our local database. Only new documents will be processed on the fly. This approach will combine the advantages of on-the-fly processing and pre-processing strategies, enabling the application to speed up searches, analyses, and reconstruction of networks. It will also facilitate implementing methods to better predict the confidence in the different interactions that are found, based for example on Bayesian networks [40].

Our tool, together with iHOP and STRING, is limited by the non-standardized nomenclature that exists in biology. Each application finds a different set of genes for each benchmarked network, with only partial overlap between the genes that are identified. Furthermore, no application finds all genes that belong to the canonical pathway defined in the KEGG server. This fact is a consequence not only of non-standard nomenclature but also of the limitations of the various datasets, where not all possible experiments and associations have been reported. Furthermore, many of these associations are reported in older papers that have yet to be made available over the web. Nevertheless, the results also emphasize the usefulness of those tools, as they tag a number of genes that interact with the benchmarked pathways but do not belong to it. The usefulness of this kind of network reconstruction will increase over time, as the nomenclature of genes and biological concepts becomes more standardized and widely used and the number of scientific documents that associate genes to biological function increases.

## Conclusions

Biblio-MetReS is a new user-friendly tool for text-based network reconstruction that is comparable in function to iHOP and STRING. Biblio-MetReS is more flexible than both, iHOP and STRING, in at least two aspects, while being equally user-friendly. First, it includes all sources of information used by iHOP and STRING, always analyzing the most up to date version of these sources. Second, the user can choose different sources of information to search from simply by checking boxes. Neither iHOP nor STRING allow for this. Furthermore, it permits analyzing the full text of scientific documents, rather than only mining the information contained in abstracts.

## Availability and Requirements

• Project name: MetReS

• Project home page: http://metres.udl.cat

• Operating system: Platform independent

• Programming language: Java

• Other requirements: Java 6.0

• License: Gnu General Public Licence

• Any restrictions to use by non-academics: none declared

## Authors' contributions

AU, XF, IT and HK carried out the database building and programming of the application. RA, FS, JV, AU & HK participated in the design of the experiments and software. AU performed the comparative studies. RA & FS conceived and coordinated the project. All authors wrote, read and approved the final manuscript.

## Endnotes

1 It must be noted that STRING has additional functionality that leads to an overestimation of the relationship between the number of citations and its usage as a text-mining tool for network reconstruction. Hereafter, all comparisons only refer to the text mining functions and results of STRING.

## Supplementary Material

Additional file 1 **Supplementary Table 1**. Benchmarking of the application. Click here for file

Additional file 2 **Supplementary Figure 1**. Representation of the number of common genes found for the different pathways in *Saccharomyces cerevisiae(1), Escherichia coli(2)*, and *Drosophila melanogaster(3) *using Biblio-MetReS, iHOP and STRING. A - Glycolysis, B - Lysine metabolism, C - RNA degradation. Click here for file

Additional file 3 **Supplementary Figure 2**. Representation of the number of common genes found for the different pathways in *Saccharomyces cerevisiae(1), Escherichia coli(2)*, and *Drosophila melanogaster(3) *using Biblio-MetReS, iHOP and STRING.A - Glycolysis, genes known to be in the pathway, B - Lysine metabolism, genes known to be in the pathway, C - RNA degradation, genes known to be in the pathway. Click here for file

Additional file 4 **Supplementary Figure 3**. Representation of the number of additional genes that are found by Biblio-MetReS as we add more data sources to Medline. Each panel shows three numbers in each square. The first number represents the number of genes found for glycolysis. The second number shows the number of genes found for lysine metabolism. The third number shows the number of genes found for RNA degradation. A - *Homo sapiens*. B - *Escherichia coli*. C - *Saccharomyces cerevisiae*. D - *Drosophila melanogaster*. In this figure we represent only the genes that are known to belong to the canonical pathways as defined in KEGG. Click here for file

Additional file 5 **Supplementary Figure 4**. Representation of the number of additional genes found by Biblio-MetReS that are known to belong to the canonical pathways under analysis as we add more data sources to Medline. Each panel shows three numbers in each square. The first number represents the number of genes found for glycolysis. The second number shows the number of genes found for lysine metabolism. The third number shows the number of genes found for RNA degradation. A - *Homo sapiens*. B - *Escherichia coli*. C - *Saccharomyces cerevisiae*. D - *Drosophila melanogaster*. In this figure we represent all genes found during the automated analysis. Click here for file
