# Supplementary material for: Biblio-MetReS: A bibliometric network reconstruction application and server
Source: BMC Bioinformatics. 2011 Oct 5;12:387. doi: 10.1186/1471-2105-12-387 (PMC3228545; doi:10.1186/1471-2105-12-387)
Supplement: Additional file 1 — Supplementary Table 1. Benchmarking of the application. [file 1471-2105-12-387-S1.PDF]

Benchmarking of the application

Organisms	Pathway	Seed genes	Benchmark 1*	Benchmark 2**
	Glycolysis	PGM1, FBA1, CDC19	iHOP vs. STRING vs. Biblio-MetReS	T1-T12
Saccharomyces cerevisiae	Lysine biosynthesis	LYS21, ARO8, LYS9	iHOP vs. STRING vs. Biblio-MetReS	T1-T12
	RNA degradation	MTR3, MPP6, CAF16, RRP41	iHOP vs. STRING vs. Biblio-MetReS	T1-T12
	Glycolysis	PGM1, ALDOA, PKLR	iHOP vs. STRING vs. Biblio-MetReS	T1-T12
Homo sapiens	Lysine biosynthesis	AADAT, AASDH, AASS	iHOP vs. STRING vs. Biblio-MetReS	T1-T12
	RNA degradation	MTR3, MPP6, CNOT4, RRP41	iHOP vs. STRING vs. Biblio-MetReS	T1-T12
	Glycolysis	pgm, fbaB, pykF	iHOP vs. STRING vs. Biblio-MetReS	T1-T12
Escherichia coli	Lysine biosynthesis	thrA, dapB, dapF	iHOP vs. STRING vs. Biblio-MetReS	T1-T12
	RNA degradation	rppH, rhlE, rnr	iHOP vs. STRING vs. Biblio-MetReS	T1-T12
	Glycolysis	Pgm, Ald, PyK	iHOP vs. STRING vs. Biblio-MetReS	T1-T12
Drosophila melanogaster	Lysine degradation	Lkr, CG9547, Gpp	iHOP vs. STRING vs. Biblio-MetReS	T1-T12
	RNA degradation	Rrp42, Mpp, Cnot4, Rrp41	iHOP vs. STRING vs. Biblio-MetReS	T1-T12

* In this benchmark we have compared the ability of three different servers to reconstruct the molecular networks regulating three types of well characterized cellular processes. We have run Biblio-MetReS using only the Medline database and used iHOP and STRING to reconstruct the networks.

** In this benchmark we have compared the differences in equivalent networks reconstructed using different information sources from the same starting set of genes. T1 – Reconstruction using general search engines. We neither discuss nor show results for these benchmark tests, because they are very non-specific. T2 – Reconstruction using Medline abstracts. T3 – Reconstruction using Medline abstract and Biomed central documents. T4 – Reconstruction using Medline abstracts and PLoS documents. T5 – Reconstruction using Medline abstracts and documents from SCOPUS. T6 – Reconstruction using Medline abstracts and documents from the Highwire database. T7 – Reconstruction using Medline abstracts and documents from Biomed central and PLoS. T8 – Reconstruction using Medline abstracts and documents from the SCOPUS and HIGHWIRE databases. T9 – Reconstruction using Medline abstracts and documents from Pubmed, PLoS and Biomed central. T10 – Reconstruction using Medline and documents from PLoS, Biomed central, Pubmed, SCOPUS and HIGHWIRE. T11 – Reconstruction using documents from all the databases in the Journals pane of Biblio-MetReS. T12 – Reconstruction using all information sources from the scientific literature and journals panes of Biblio-MetReS.
