# Supplementary figures and images for: Biblio-MetReS: A bibliometric network reconstruction application and server
Source: BMC Bioinformatics. 2011 Oct 5;12:387. doi: 10.1186/1471-2105-12-387 (PMC3228545; doi:10.1186/1471-2105-12-387)

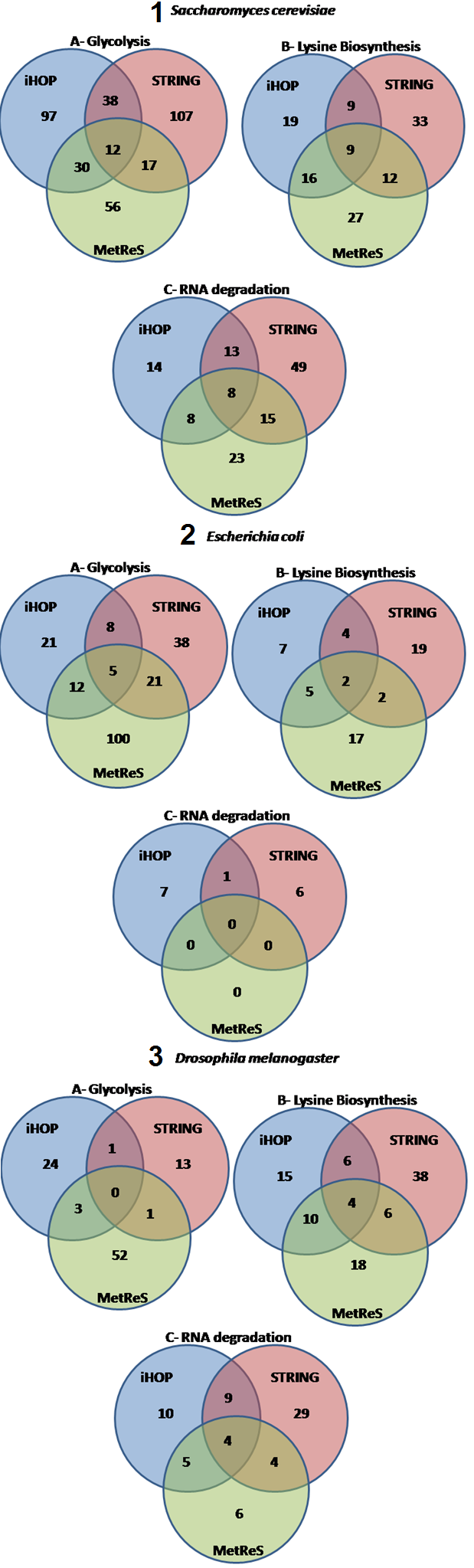

Supplement: Additional file 2 — Supplementary Figure 1. Representation of the number of common genes found for the different pathways in Saccharomyces cerevisiae(1), Escherichia coli(2), and Drosophila melanogaster(3) using Biblio-MetReS, iHOP and STRING. A - Glycolysis, B - Lysine metabolism, C - RNA degradation. [file 1471-2105-12-387-S2.PNG]

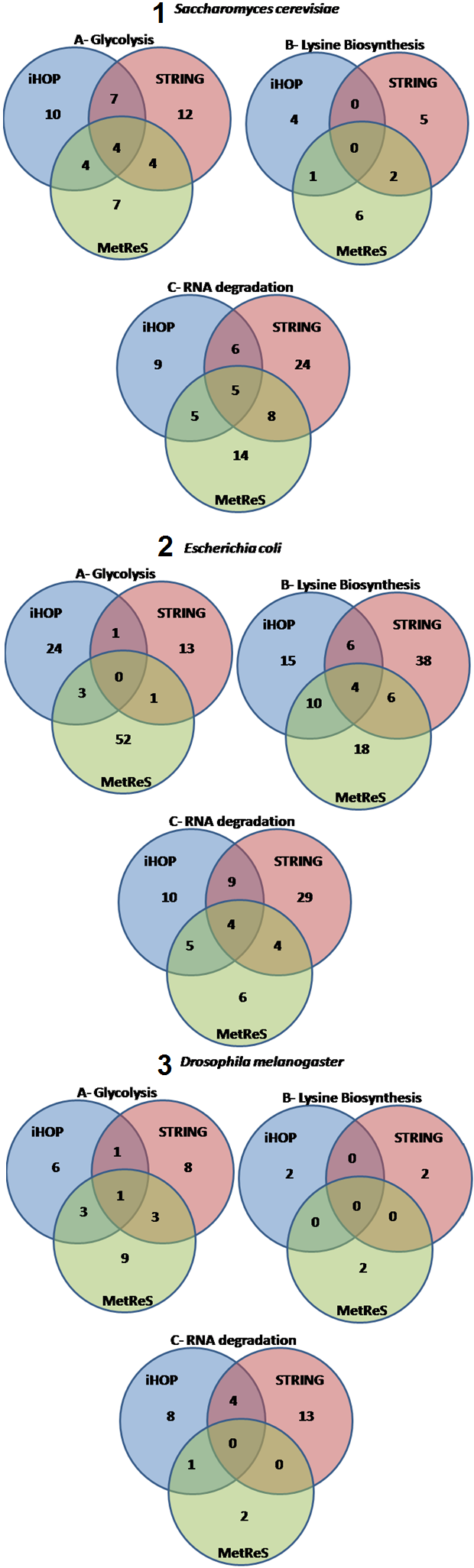

Supplement: Additional file 3 — Supplementary Figure 2. Representation of the number of common genes found for the different pathways in Saccharomyces cerevisiae(1), Escherichia coli(2), and Drosophila melanogaster(3) using Biblio-MetReS, iHOP and STRING.A - Glycolysis, genes known to be in the pathway, B - Lysine metabolism, genes known to be in the pathway, C - RNA degradation, genes known to be in the pathway. [file 1471-2105-12-387-S3.PNG]

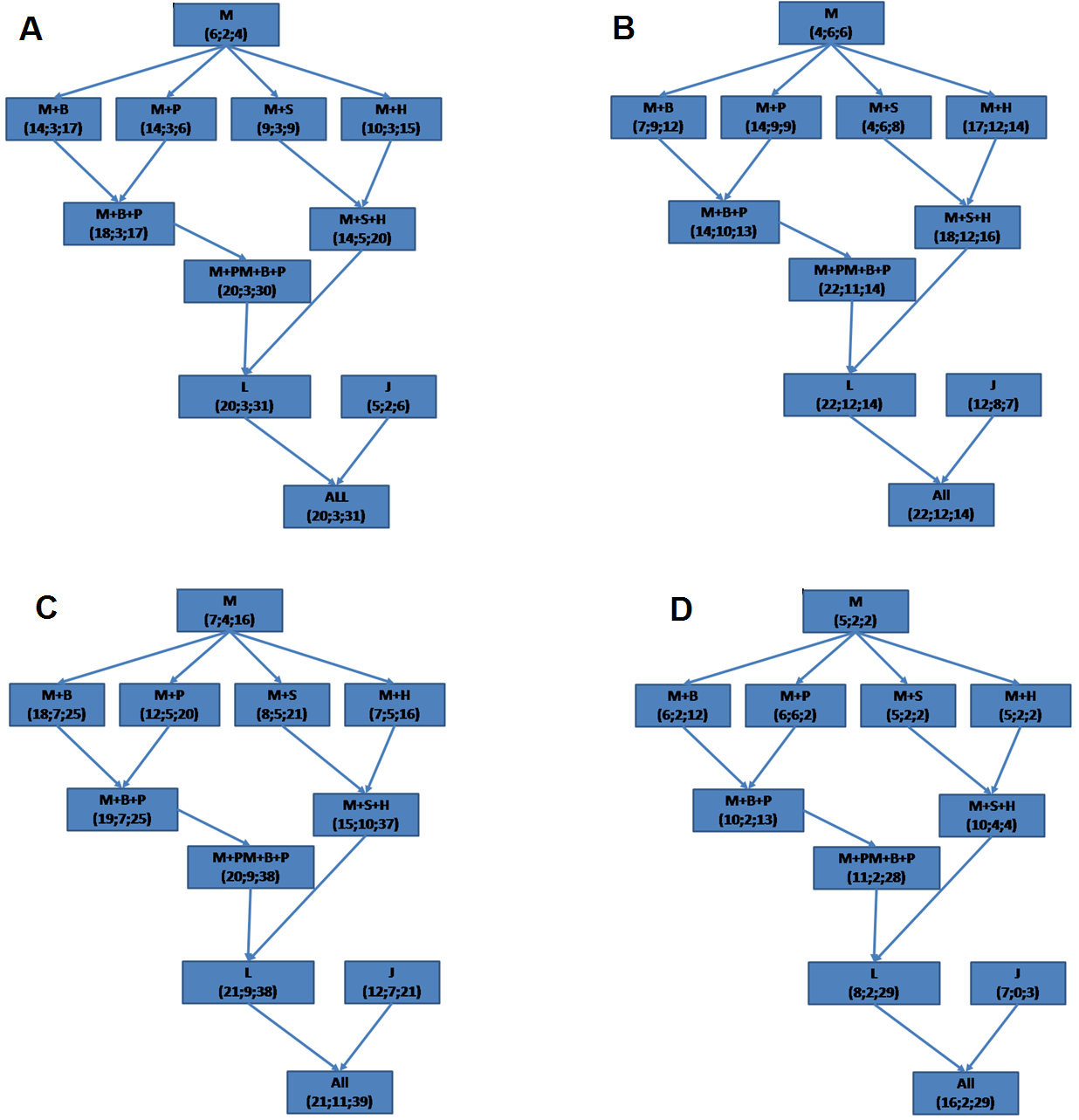

Supplement: Additional file 4 — Supplementary Figure 3. Representation of the number of additional genes that are found by Biblio-MetReS as we add more data sources to Medline. Each panel shows three numbers in each square. The first number represents the number of genes found for glycolysis. The second number shows the number of genes found for lysine metabolism. The third number shows the number of genes found for RNA degradation. A - Homo sapiens. B - Escherichia coli. C - Saccharomyces cerevisiae. D - Drosophila melanogaster. In this figure we represent only the genes that are known to belong to the canonical pathways as defined in KEGG. [file 1471-2105-12-387-S4.PNG]

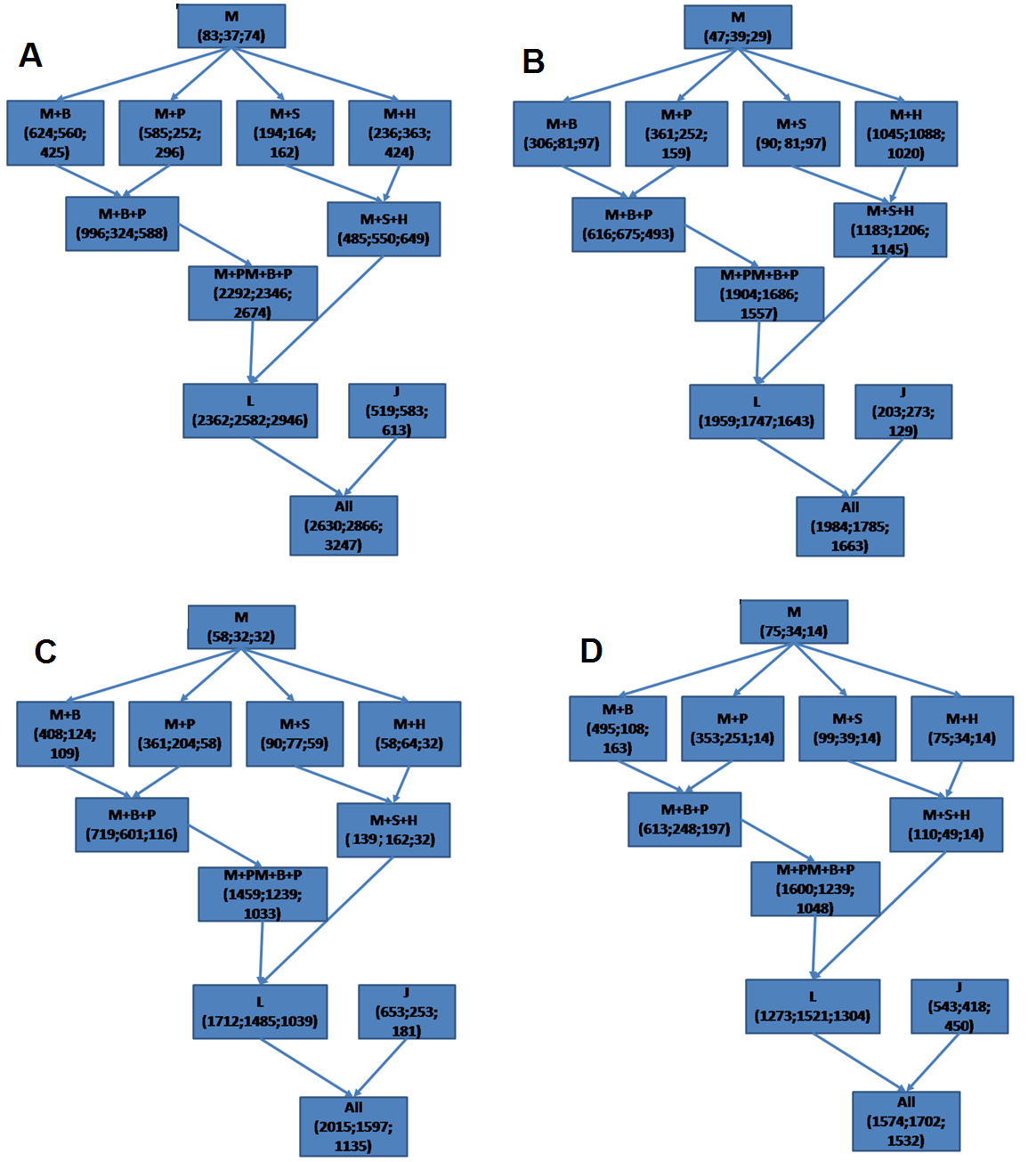

Supplement: Additional file 5 — Supplementary Figure 4. Representation of the number of additional genes found by Biblio-MetReS that are known to belong to the canonical pathways under analysis as we add more data sources to Medline. Each panel shows three numbers in each square. The first number represents the number of genes found for glycolysis. The second number shows the number of genes found for lysine metabolism. The third number shows the number of genes found for RNA degradation. A - Homo sapiens. B - Escherichia coli. C - Saccharomyces cerevisiae. D - Drosophila melanogaster. In this figure we represent all genes found during the automated analysis. [file 1471-2105-12-387-S5.PNG]
